# Review: Capripoxvirus Diseases: Current Status and Opportunities for Control

**DOI:** 10.1111/tbed.12444

**Published:** 2015-11-13

**Authors:** E. S. M. Tuppurainen, E. H. Venter, J. L. Shisler, G. Gari, G. A. Mekonnen, N. Juleff, N. A. Lyons, K. De Clercq, C. Upton, T. R. Bowden, S. Babiuk, L. A. Babiuk

**Affiliations:** ^1^ Department of Veterinary BiosciencesFaculty of Veterinary MedicineUniversity of HelsinkiHelsinkiFinland; ^2^ Department of Veterinary Tropical DiseasesUniversity of PretoriaPretoriaSouth Africa; ^3^ Department of MicrobiologyUniversity of IllinoisUrbanaILUSA; ^4^ National Animal Health Diagnostic and Investigation Center (NAHDIC)SebetaEthiopia; ^5^ Bill & Melinda Gates FoundationSeattleWAUSA; ^6^ The Pirbright InstitutePirbrightUK; ^7^ European Commission for the Control of Foot‐and‐Mouth DiseaseFood and Agriculture Organisation of the United NationsRomeItaly; ^8^ CODA‐CERVAVesicular and Exotic Diseases UnitUccleBelgium; ^9^ Department of Biochemistry and MicrobiologyUniversity of VictoriaVictoriaBCCanada; ^10^ CSIROHealth & Biosecurity FlagshipAustralian Animal Health LaboratoryGeelongVic.Australia; ^11^ Canadian Food Inspection AgencyNational Centre for Foreign Animal DiseaseWinnipegWACanada; ^12^ University of AlbertaEdmontonABCanada

**Keywords:** lumpy skin disease, sheeppox, goatpox, immunity, control, vaccines

## Abstract

Lumpy skin disease, sheeppox and goatpox are high‐impact diseases of domestic ruminants with a devastating effect on cattle, sheep and goat farming industries in endemic regions. In this article, we review the current geographical distribution, economic impact of an outbreak, epidemiology, transmission and immunity of capripoxvirus. The special focus of the article is to scrutinize the use of currently available vaccines to investigate the resource needs and challenges that will have to be overcome to improve disease control and eradication, and progress on the development of safer and more effective vaccines. In addition, field evaluation of the efficacy of the vaccines and the genomic database available for poxviruses are discussed.

## Introduction

Lumpy skin disease (LSD), sheeppox (SPP) and goatpox (GTP) are economically important capripoxvirus (CaPV) diseases of domestic ruminants with substantial impact on the livelihoods of small‐scale farmers and poor rural communities in endemic regions.

LSD, SPP and GTP are known to be present in Syria and Iraq. Since 2011, conflicts in these countries have promoted factors such as mass movement of refugees and farm animals, collapsed veterinary services and lack of available vaccines and medicines, leading to delayed or failed containment of epidemics of many infectious human and veterinary diseases.

In 2015, millions of civilians are looking for safety in Turkey, Jordan, Lebanon and Iran, being relocated to temporary settlements or dispersed among local communities. The need to support such a large number of people, in addition to the local population, puts these countries under immense economic pressure. Refugees may also travel with unvaccinated cattle, sheep and goats. As an example, in Lebanon there are currently 1.2 million registered Syrian refugees and a 60% increase in the quantity of livestock near the Syrian border (ProMed 20150527.3389044). The movement of farm animals without proper health checks has been associated with the current spread of LSD virus (LSDV), SPP virus (SPPV) and GTP virus (GTPV) in the Middle and Near East (ProMed 20150410.3290468).

As vaccination of cattle and small ruminants in the war‐torn areas is neither possible nor safe to perform, it is highly likely that the conflict regions will continue to serve as a source of infection, until reconstruction of basic infrastructure can be commenced. In addition, in any of those countries where LSD, SPP or GTP are currently endemic, culling of all infected and in‐contact animals is not an affordable or feasible disease control strategy.

It is also well known that CaPV diseases are extremely difficult to control using only total or modified stamping‐out, animal movement restrictions and quarantine. However, the experiences obtained from Israel and the northern part of Cyprus show that LSD outbreaks can be successfully contained by a well‐organized vaccination campaign, using sufficient coverage and effective vaccines.

Until recently, very little interest in CaPV research has been shown outside endemic regions, and funding opportunities have been scarce for these neglected infectious diseases. However, the significant emergence of CaPV in the Middle and Near East and the reported problems associated with the use of different CaPV vaccines, illustrate the pressing need for improved control strategies and have escalated CaPVs as a research priority. This shift also provides an opportunity to reassess control strategies and identify new opportunities for smallholder farmers who rely on livestock for their livelihoods and therefore have the greatest need for improved disease control.

The aim of this review is to highlight the current epidemiological status of CaPVs, scrutinize the vaccines available in affected regions and to investigate the resources and challenges facing CaPV control and the attempts to further develop safer and more effective vaccines.

### Geographical distribution

LSD is widespread throughout Africa, causing particularly severe outbreaks in the Horn of Africa. Prior to 2012, only sporadic LSDV outbreaks were reported in the Middle East region. In the summer of 2012, LSD was reported by the Israeli veterinary authorities in beef herds in the northern parts of the Golan Heights, adjacent to the borders of Syria (ProMed 20120728.1218484). The primary source of infection was inconclusive, although the outbreak locations indicated that LSDV was likely to be circulating in the cattle populations in Syria. Between 2012 and 2013, the disease spread throughout the northern half of Israel, infecting both beef and dairy herds (Ben‐Gera et al., 2015). In late 2012, LSD was detected in Lebanon (ProMed 20130118.1505118) where 34 outbreaks were reported, followed by outbreaks in Jordan (ProMed 20130612.1768278) (Abutarbush et al., 2013) and the West Bank (ProMed 20130311.1581763).

**Figure 1:**
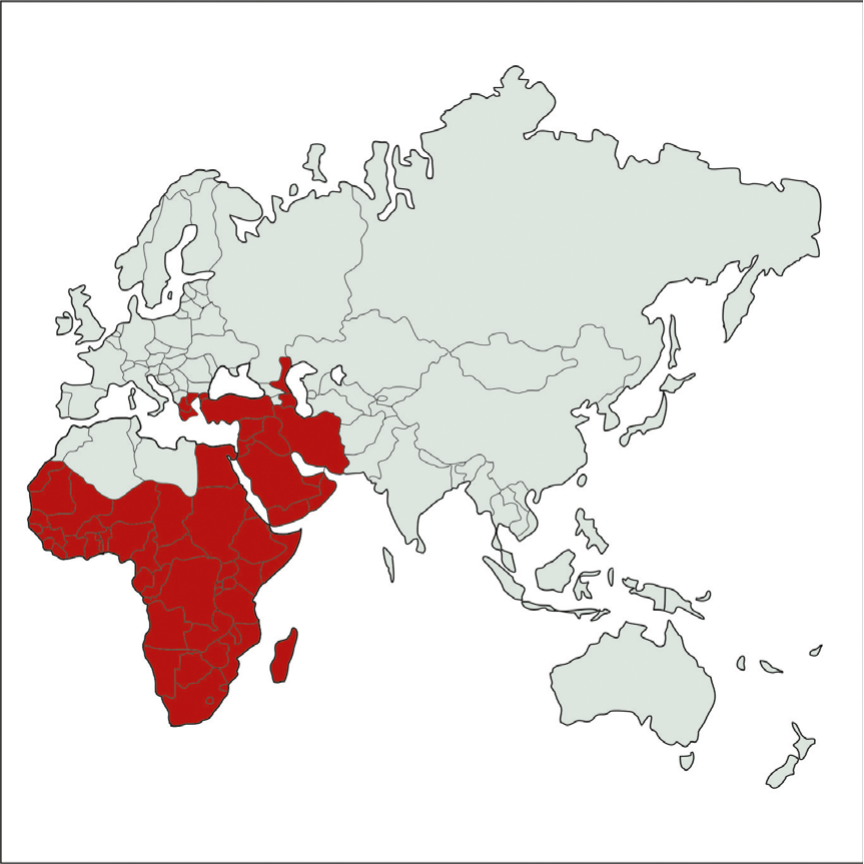
Geographical distribution of lumpy skin disease. [Colour figure can be viewed at wileyonlinelibrary.com]

Between 2013 and 2015, LSDV spread throughout Turkey (ProMed 20130831.1915595) to the extent where LSD may now become endemic in the country. Incursion of the virus was subsequently reported in Iraq (ProMed 20130718.1831781) (Al‐Salihi and Hassan, 2015) and along the western borders of Iran (ProMed 20140623.2561202). Surprisingly, the virus seems to be capable of spreading over long distances, as in 2014 the disease was reported in Azerbaijan (ProMed 20140719.2621294). The distance between Syria and the northern peninsula of Cyprus is only approximately 60 km and the first cases of LSD were reported there in late 2014 (ProMed 20141205.3012426). It is highly likely that the incursions of LSDV in Iraq, Cyprus and Azerbaijan were associated with unauthorized cattle movements (Al‐Salihi and Hassan, 2015). The local veterinary authorities in the northern part of Cyprus also identified imported hay and straw from Turkey as a potential source of LSDV. Kuwait reported outbreaks in late 2014 and early 2015 (ProMed 20150206.3147602) and Saudi Arabia in spring 2015 (ProMed 20150430.3333997). In August 2015, the first incursion of LSDV was reported in the European Union territory in Greece, close to the river Evros and the Turkish border (OIE Wahid, ProMed 20150821.3594203) and in September in the northern Caucasus region of Russia, Dagestan and Chechnya (OIE Wahid, ProMed 20150904.3622855 and 20150921.3659823) (Fig. 1). LSD is clearly on the move, increasing the disease risk in Bulgaria, Macedonia, Albania, Georgia, Armenia, Turkmenistan, Afghanistan and Pakistan.

**Figure 2:**
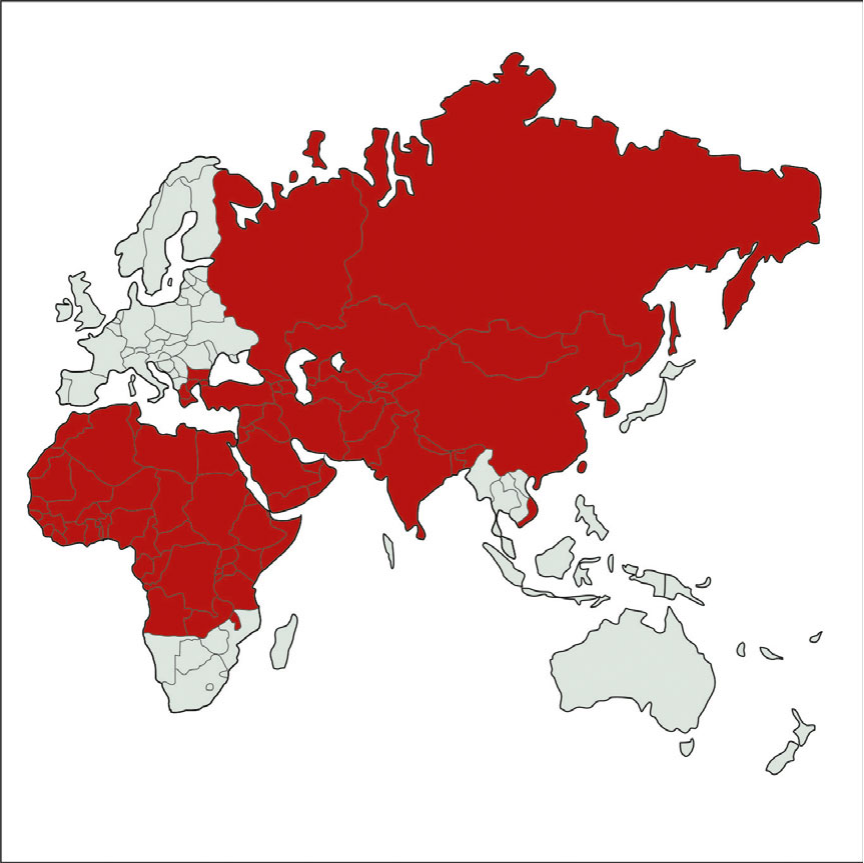
Geographical distribution of sheeppox and goatpox. [Colour figure can be viewed at wileyonlinelibrary.com]

Historically, the global distribution of SPP and GTP has been wider than LSD. Indeed, cases of SPP and GTP regularly occur in northern and central Africa, across the Middle East and the Indian subcontinent, Iran, Iraq, Russia, Kazakhstan, Kyrgyzstan, Afghanistan, Pakistan, Nepal, Mongolia, China, Bangladesh, Vietnam and Chinese Taipei (OIE WAHID). The diseases are also endemic in Turkey and between 2013 and 2015 four outbreaks occurred in Bulgaria and several outbreaks were reported in Greece (OIE WAHID) (Fig. 2). According to the OIE WAHID‐database, the incidence of SPP in Greece is still continuing in 2015 despite implementation of an extensive stamping‐out policy.

### Economic impact

LSD, SPP and GTP are categorized by the OIE as notifiable diseases due to their potential for rapid spread and substantial economic impact. Cattle are particularly susceptible to LSD during peak lactation, which together with secondary mastitis and prolonged high fever, affects milk production. Abortions and temporary or permanent infertility occur among infected animals. Emaciation and a long convalescence period can significantly decrease the growth rate in beef cattle (Weiss, 1968). Furthermore, deep pox lesions in the skin leave permanent scars, decreasing the value of skins and hides for use in the leather industry (Green, 1959). In intensive dairy cattle farming units in the Middle East, total direct production losses caused by LSD were estimated to be 45–65% (Somasundaram, 2011). For SPP and GTP, the direct animal losses and the decreased productivity of surviving animals cause average annual losses in income of 30–43% and flocks or herds can take up to 6 years to recover from an outbreak (Garner and Lack, 1995). The mortality rate of SPP and GTP can sometimes be considerably high, particularly amongst lambs and kids. At a national level, restrictions on international trade of live animals and animal products, costly vaccination campaigns and compulsory limitations of animal movements can cause significant indirect financial losses. The poorest smallholder farmers and pastoralists whose income and wellbeing rely mostly on their livestock and sale of milk, animals, hides and manure, bear the heaviest burden during outbreaks.

LSD, SPP and GTP have been identified as one of the major impediments for genetic improvement of cattle, sheep and goat populations and, consequently, for the development of intensive production units in Africa, the Indian subcontinent and Asia. It is well known that high‐producing dairy cattle, such as Holstein‐Friesian (HF) and Jersey, as well as European breeds of sheep and goats are more susceptible to CaPV infection than indigenous African and Asian cattle, sheep and goat breeds (Davies, 1991a; Bhanuprakash et al., 2006). The susceptibility of European cattle breeds and challenges facing dairy‐genetics improvement in LSDV‐endemic settings in Ethiopia was recently highlighted by Gari et al. (2011). The average duration of the lactation period of local zebu cows was shorter (240 days) than for HF/cross breeds (305 days) and for zebu cattle the milk take‐off per lactation was significantly lower (323 l) than for HF/cross cows (3694 l). In this study comprising a selection of farms, the HF and Jersey breeds were found to be almost three times more susceptible for LSDV than zebus (annual cumulative incidence of LSDV infection of 33.93% and 13.41% respectively) and the annual mortality rates due to LSDV infection of zebus was considerably lower than for HFs (1.26% compared to 7.43%). In addition, estimated total production losses in infected cattle due to decrease in milk and beef production, loss of draft power, mortality, veterinary treatments and vaccination costs were estimated to be 6.43 USD per head for local zebu and 58 USD per head for HF/cross breeds (Gari et al., 2011).

### Epidemiology

For LSDV infection in cattle, morbidity rates can vary from 5 to 45% whereas the mortality rate usually remains below 10%, although both can be considerably higher (morbidity sometimes up to 100%) when incursion occurs in European cattle breeds (Coetzer, 2004). Highly contagious SPPV and GTPV are able to cause very high morbidity (70–90%) and mortality up to 50%. Young animals show more severe disease, and mortality in lambs and kids may be as high as 100% (Rao and Bandyopadhyay, 2000). The virulence of different CaPVs may vary but the severity of the clinical disease more often depends on the species, breed, age, immune status and stage of production of the host. Historically, CaPVs were considered to be host‐specific. In general SPPV and GTPV cause more severe clinical disease in sheep or goats, although some strains can affect both species. Surprisingly, a recent study in Ethiopia revealed that GTPV was solely responsible for all investigated outbreaks in both sheep and goats throughout the study (Gelaye et al., 2015). So far no evidence of SPPV or GTPV in wild ruminants exists. In contrast, LSDV infects domestic cattle and Asian water buffalo (Ali and Obeid, 1977; El‐Nahas et al., 2011), and some strains may also replicate in sheep and goats. For LSDV it is not known where the virus resides during the time of minimal or no vector activity. Some wild ruminants, such as springbok (Le Goff et al., 2009; Lamien et al., 2011a), impala and giraffe (Young et al., 1970), are known to be susceptible to the virus and African buffaloes have been found to be seropositive (Davies, 1982; Fagbo et al., 2014). In addition, LSDV‐specific antibodies have been demonstrated in various wild ruminants such as blue wildebeest, eland, giraffe, impala and greater kudu (Barnard, 1997). However, the role of wildlife in the epidemiology of LSD is not well understood. Davies (1982) suggested that in Kenya the virus is maintained in the forest or at the forest edge locations among wild buffalo populations in moderate rainfall zones at 1000–2500 m altitude (Davies, 1982).

There are no reports describing a carrier state for CaPV infected animals. Some innate resistance to LSDV is known to occur in cattle (Weiss, 1968). This also has been demonstrated using experimentally inoculated cattle: only 50% are likely to develop clinical signs although all the animals become viraemic (Tuppurainen et al., 2005; Osuagwuh et al., 2007; Annandale et al., 2010). It has been suggested that sporadic clinical cases that occur during most years in endemic regions may be associated with a maintenance cycle in asymptomatic cattle (Davies, 1982). Mechanical transmission has been shown to occur from the healthy looking skin of viraemic cattle to naïve hosts by blood‐feeding tick vectors (Tuppurainen et al., 2013a).

### Transmission

Sheep and goats can be infected experimentally via intradermal inoculation and oral or intranasal administration of SPPV and GTPV (Bowden et al., 2008). Infected sheep and goats shed virus in oral, nasal and ocular secretions and transmission occurs through aerosols and direct contact (Kitching and Mellor, 1986; Bowden et al., 2008). Due to the stability of the virus, SPPV and GTPV may persist in the environment for prolonged periods of time, leading to infection of naïve animals. Unlike LSDV, insect vectors are not required for the transmission of SPPV and GTPV, although due to high viral loads in the skin, mechanical transmission may occur by insect vectors. *Stomoxys calcitrans* (stable fly) has been shown experimentally to transmit SPPV and GTPV; whereas *Mallophaga* species, *Damalinia* species, *Hydrotaea irritans* and *Culicoides nubeculosus* were not able to transmit SPPV despite the virus being isolated from *Hydrotaea irritans* after feeding on infected sheep (Kitching and Mellor, 1986). These studies indicate that as insect vectors may play a role under experimental conditions, the role of insect vectors in the field remains unclear. Outbreaks of SPP and GTP may occur throughout the year, supporting the importance of non‐vector transmission pathways.

It is currently believed that the main mode of transmission of LSDV is mechanical via blood‐feeding insects with frequent feeding habits. The most important vector is likely to vary between affected regions, depending on the climate, season, environmental temperature, humidity and vegetation, favouring different insect and tick species. The relative importance of vectors may also vary within a region as changes in climate may affect the local arthropod populations and viral spread. Weather changes, such as cold spells that adversely affect insect vector populations, also reduce LSDV transmission (Davies, 1982, 1991b). Although LSDV outbreaks are more common in warm and humid weather conditions, they also occur during the dry season and winter months (Haig, 1957; Nawathe et al., 1982). The impact of global climate change and the evidence that insect vectors play a role in the transmission of LSDV suggest there are real risks of LSD establishing in the Middle East and Asia, as well as the further spread of the disease into other geographical regions.

Live LSDV was isolated from *Stomoxys calcitrans* and *Biomyia fasciata* flies after feeding on infected cattle (Weiss, 1968). Experimentally, the female *Aedes aegypti* mosquito has been shown to transmit LSDV from infected to susceptible cattle (Chihota et al., 2001). However, attempts to transmit the virus by the *Anopheles stephensi* mosquito, stable flies and *Culicoides nubeculosus* biting midges were not successful (Chihota et al., 2003). It has been postulated that biting flies have to feed on skin lesions to obtain enough virus for transmission to occur (Carn and Kitching, 1995a).

The first LSDV outbreak in Sudan was associated with the presence of *Amblyomma* ticks on affected animals (Ali and Obeid, 1977). Recently, Tuppurainen et al. (2011) reported the potential role of ixodid ticks in the transmission of LSDV. Transovarial transmission of LSDV by *Rhipicephalus (Boophilus) decoloratus*, mechanical or intrastadial transmission by *Rhipicephalus appendiculatus* and *Amblyomma hebraeum* males, as well as trans‐stadial transmission by *Amblyomma hebraeum* have now been reported (Lubinga et al., 2013, 2014; Tuppurainen et al., 2013a,b).

Direct contact between infected and susceptible animals is considered an inefficient route of transmission for LSDV (Weiss, 1968; Carn and Kitching, 1995b). Transmission was, however, achieved when naïve cattle were allowed to share a drinking trough with severely infected animals in insect‐free facilities (Haig, 1957). Infected animals start to excrete the virus in saliva, as well as ocular and nasal discharges, soon after the onset of clinical signs (Haig, 1957; Babiuk et al., 2008). Therefore, the animals may become infectious in the early stages of the disease and further investigations of transmission through direct contact are required. Cows infected with LSDV are known to give birth to calves with pox lesions in the skin through vertical disease transfer. The disease is rarely transmitted to suckling calves through infected milk or from the skin lesions in the teats (Weiss, 1968).

LSD virus is known to persist in the male genital tract and viral DNA has been found in semen for 5 months after infection (Irons et al., 2005; Tuppurainen et al., 2005; Bagla et al., 2006; Annandale et al., 2010). In a recent study, Annandale et al. (2013) showed experimentally that seminal transmission of LSDV in cattle is possible Whether this also occurs during natural mating or artificial insemination is not known. Immunization of experimental bulls using LSDV vaccine prevented shedding of LSD in semen (Osuagwuh et al., 2007), it is however not clear if standard stepwise washing of embryos would successfully eliminate the virus (Bielanski, 2007).

Recently, Klausner et al. (2017) investigated the potential role of air currents in a long‐distance dispersal of LSDV contaminated insects during the 1989 and 2006 LSDV outbreaks in Israel.

### Immunity against CaPV and experience obtained from orthopoxvirus research

Susceptibility of the host to CaPVs depends on several factors, including the virulence of the virus and the immune status, age and breed of the host. A natural resistance to LSDV infection, not associated with immunity, is known to occur in cattle and subclinical LSDV infections are common in the field (Weiss, 1968). Typically, in experimentally infected cattle approximately one‐third show no clinical signs at all, although all of the infected animals became viraemic (Tuppurainen et al., 2005; Osuagwuh et al., 2007; Annandale et al., 2010). The presence of asymptomatic viraemic animals, which are capable of transmitting the virus via arthropod vectors, complicates control and eradication of LSDV, particularly in countries where slaughter of all infected and in‐contact animals is not feasible.

Immunity against CaPV is predominantly cell‐mediated although humoral immunity also plays a role (Kitching et al., 1987). In general, it has been believed that a replicating agent generates more broad protective immunity than a non‐replicating one. However, most recent studies have shown that also inactivated SPPV vaccines can confer a protective immunity in sheep, comparable to that provided by a live SPPV vaccine (Z. Boumart, unpublished data).

Most progeny viruses remain inside infected cells, with the exception of the extracellular enveloped virions, which are released by budding from infected cells. These may infect neighbouring cells or escape into the blood and be disseminated throughout the body. By spreading locally and directly from cell to cell, the virus is out of reach of circulating antibodies, which are able to limit the spread of the virus, but do not prevent replication of the virus at the site of inoculation (Boulter and Appleyard, 1973). In addition, it has been reported that after experimental intradermal inoculation, SPPV and GTPV were able to infect monocyte/macrophage linage cells, suggesting that these cells may aid in the systemic spread of the virus (Embury‐Hyatt et al., 2012).

Animals affected by CaPVs will eventually clear the infection and do not become carriers. CaPVs are more than 95% identical on a genome level. Thus, all CaPVs share a common major antigen for neutralizing antibodies and animals that recover from natural infection are resistant to reinfection (Kitching, 1986a). However, field experience obtained from the most recent outbreaks of LSDV in the Middle East and the Horn of Africa indicate that cross‐protection provided by non‐homologous vaccine viruses is only partial (Khalafalla et al., 1993; Yeruham et al., 1994; Somasundaram, 2011; Ayelet et al., 2013; Tageldin et al., 2014).

The role of antibodies in protection against CaPV was demonstrated by passive transfer of sera from infected sheep, which protected the recipient sheep against CaPV challenge (Kitching, 1986b), suggesting that antibodies alone are sufficient for protection. However, the immune status of a previously infected or vaccinated animal cannot be related directly to serum levels of neutralizing antibodies (Weiss, 1968; Kitching, 1986a). After vaccination, antibodies usually appear within 15 days and reach the highest level 30 days post‐inoculation, eventually decreasing below detectable levels. Vaccinated animals or those showing mild disease, may develop only low levels of neutralizing antibodies which are often below the detection limits of currently available serological tests, even though these animals would be resistant to challenge. Interestingly, virulent SPP or GTP viruses elicits antibody responses, but the attenuated KS‐1 vaccine (LSDV) does not always elicit detectable neutralizing antibodies in sheep, goats and cattle (Bowden et al., 2009) although vaccinated animals are still protected against virulent CaPV challenge. In naturally infected animals, antibodies against CaPV can usually be detected for 3–6 months after infection but further studies are required to investigate the long‐term persistence of CaPV antibodies post‐infection. In early studies by Westhuizen (1964) and sited by Weiss (1968), calves born to immunized cows had a passive immunity derived from the colostrum that persisted up to 6 months (Weiss, 1968). No recent studies have been carried out on the duration of protection provided by maternal antibodies against LSDV.

Annual vaccination against CaPV is recommended by live attenuated vaccine manufacturers, as the maximum duration of protection has been reported to be 22 months (Kitching, 2003).

Because CaPVs belong to the same virus family (*Poxviridae*) as variola virus and vaccinia virus (VACV), prior research with the smallpox vaccine may benefit the CaPV vaccine field. For orthopoxviruses humoral responses are considered to be sufficient for protection against re‐infection, whereas both humoral and cellular responses are required for clearance of a primary orthopoxvirus infection (Sette et al., 2009; Moutaftsi et al., 2010; Moss, 2011). Early studies show that neutralizing antibodies with a titre above 1 : 32 correlated with protection against smallpox (Downie and McCarthy, 1958; Mack et al., 1972).

VACV encodes approximately 200 proteins, and there are nine VACV‐specific B‐cell epitopes (Moutaftsi et al., 2010). Experimental evidence shows at least five of these proteins (H3, A27, B5, D8 and L1; VACV‐Copenhagen nomenclature) elicit protective neutralizing antibodies in mice (Rodriguez et al., 1985; Gordon et al., 1991; Wolffe et al., 1995; Galmiche et al., 1999; Hsiao et al., 1999), and one protein (A33) induces a protective, but non‐neutralizing antibody response. For example, anti‐B5 antibodies detect enveloped viruses, and vaccination of animals with the B5 protein is sufficient to protect against a lethal intranasal dose of VACV in mice (Galmiche et al., 1999). Yet another report shows that a smallpox DNA vaccine, consisting of the A33R, A36R, L1R and B5R genes is sufficient to provide protection against a lethal challenge of monkeypox virus (Hooper et al., 2003; Heraud et al., 2006). These neutralizing antibodies against VACV proteins are effective because these proteins are highly conserved across members of the genus *Orthopoxvirus*. Whether vaccination with the CaPV homologues of these antigenic VACV proteins are similar enough to each other that inoculation with proteins from one member of the genus *Capripoxvirus* would raise a protective response against the other members of the genus remains to be investigated. It should be noted that the B5R and A33R genes of orthopoxviruses and CaPV share very low sequence similarity. The L1 protein is the most conserved between orthopoxviruses and CaPV. Reviewing the alignments of these proteins indicates that the similarity between the three CaPV proteins is very close to the similarity between VACV and variola virus, which suggests that there could be cross‐protection between the CaPV epitopes. Sequencing of more CaPV genomes would help to solidify this prediction. However, because there is such a large divergence between the CaPV and orthopoxviruses, it is not possible to predict whether the CaPV orthologues will also elicit neutralizing antibodies.

T‐cells are also important for the protective effects elicited by smallpox vaccines. A robust B‐cell response cannot occur in the absence of CD4+ T‐cells. Over 100 VACV epitopes for MHC class I molecules and more than 40 MHC class II epitopes have been described in humans (Sette et al., 2009; Moutaftsi et al., 2010). These epitopes have been identified by (i) bioinformatics analyses (Immune Epitope Database and Analysis Resource; IEDB), (ii) studies that include approaches that identified polypeptide fragments of VACV proteins that stimulate VACV‐specific CD8+ T cells and (iii) isolating VACV‐derived peptides from MHC molecules (Jing et al., 2005; Tscharke et al., 2005; Golden and Hooper, 2008; Moutaftsi et al., 2010). Very little is known about the role of CD4+ and CD8+ T‐cells for protective immunity against natural SPPV, GTPV and LSDV infections and these responses could be investigated by using, for example, the approaches discussed above. Such results would assist in developing rapid diagnostic assays or assays for quality control of vaccine production.

The percutaneous route of smallpox vaccination produces neutralizing antibodies in 83% of patients, when compared with cutaneous vaccination, which produces neutralizing antibodies in 23% of the vaccinates (Galasso et al., 1977; McClain et al., 1997). For practical reasons vaccines against CaPV are given via a subcutaneous route in the field. Whether this is the most efficient route for immunization is unknown.

Modified vaccinia virus Ankara (MVA) was attenuated by serially passaging the wild‐type VACV‐Ankara in chicken embryo fibroblasts to create a virus that was no longer capable of replicating in human cells (Mayr et al., 1975). MVA is safer, but requires a higher dose of virus inoculum, than replication‐competent VACV for protection against a lethal infection in laboratory animals. MVA also lacks many immune evasion genes otherwise present in VACV (Antoine et al., 1998), suggesting that the absence of these genes may also shape the protective immune response or modulate virulence. The different CaPV vaccines were developed in a manner similar to MVA. However, the genetic make‐up, the immunogenicity and pathogenicity of many of these attenuated viruses has not been determined in controlled laboratory experiments, making it difficult to determine which of the currently used vaccines are most effective.

### Control and eradication

Successful control and eradication of SPPV and GTPV relies heavily on early detection of the index case, rapid implementation of stamping‐out of all infected and in‐contact animals, strict movement control, quarantine and disinfection. In those areas where the disease is newly introduced, early detection requires disease awareness amongst field veterinarians, farmers and animal care staff, as well as diagnostic capacity of the local laboratories. As LSDV is transmitted by arthropod vectors, it would probably be more difficult to eliminate. Any delay in stamping‐out of infected animals would give time for vectors to become contaminated and transmit the disease. Due to asymptomatic but vireamic animals, killing only those cattle or water buffaloes showing clinical signs of LSD (known as ‘modified stamping out’) is unlikely to be effective alone, although it may have benefits combined with other control measures.

Vaccination is the most effective way to control the spread of CaPVs. Only live attenuated vaccines are currently available against LSDV, SPPV and GTPV. These vaccines are cheap (currently € 1.5–2.0 per dose) and provide good protection if sufficient herd immunity (over 80%) is maintained by carrying out annual vaccinations. As an example, the LSD outbreaks in Israel in 2012–13 (Ben‐Gera et al., 2015) and in the northern part of Cyprus in 2014–15 were successfully controlled by mass vaccination, using a LSDV vaccine.

Lack of compulsory and consistent vaccination strategies together with ineffective animal movement control are the most common causes for the uncontrollable spread of CaPV. Transhumance and nomadic farming practices, common in CaPV endemic regions complicates disease control, and vaccination of animals moving over long distances should be a priority. In some areas, farmers have used vaccines obtained from black markets (Abutarbush, 2014). However, the use of unauthorized vaccines should be avoided as they are often unlabelled and the real identity and titre of the vaccine virus is unknown. They may also have been diluted or contaminated with adventitious pathogens and vaccine vials may have been inappropriately stored or be out of date.

Prior to the use of currently available SPPV vaccines against LSDV in cattle, the efficacy of the vaccine should be demonstrated, using challenge experiments. As the geographical range and host species of peste des petits ruminants (PPR) virus, SPPV and GTPV are identical, ideally eradication of all three diseases, through mass vaccination should be co‐ordinated.

### Vaccines currently available

None of the live attenuated CaPV vaccines are authorized for use in non‐endemic countries. The use of an SPPV vaccine against LSDV has been restricted to those countries where SPP, GTP and LSD overlap, such as central and northern Africa, the Middle East, Turkey, Iraq and Iran. The Yugoslavian RM65 SPPV vaccine, at a 10 times higher dose than indicated for sheep, has commonly been used for cattle across the Middle East. In Egypt both the Romanian SPP and Kenyan sheep and goat pox (KSGP) virus vaccines have been used for cattle (Davies, 1991a; Brenner et al., 2009; Somasundaram, 2011; Abutarbush, 2014). The Bakirköy SPPV (at three to four times the recommended dose for sheep) has been used in Turkey against LSDV. Using molecular methods, the real identity of the KSGP virus O‐240 and O‐180 strains has been shown to be LSDV and not SPPV or GTPV (Tulman et al., 2002; Lamien et al., 2011b; Tuppurainen et al., 2014), and use of these strains is not recommended for cattle before their safety and efficacy are evaluated using challenge experiments in a controlled environment.

Several locally produced SPP and GTP vaccines are available against SPPV and GTPV, particularly in the Indian subcontinent. The KSGPV O‐240, O‐180, as well as the RM65 strains are commonly used against SPPV in the Middle East, whereas the Bakirköy SPPV vaccine is used in Turkey. The attenuated Gorgan and Mysore GTPV strains are used against GTPV (Kitching, 2003). Currently, no vaccines with a Differentiation of Infected from Vaccinated Animals (DIVA)‐component are commercially available against CaPVs. All the currently used vaccines are manufactured using primary cells, which make quality assurance difficult and can cause issues with endogenous agents. Although there are cell lines available to grow CaPV such as OA3.Ts (Babiuk et al., 2007), they are not licensed for vaccine production use.

Lumpy skin disease has been endemic in South Africa for decades and vaccination against LSDV is a common practice. Only cattle are vaccinated and vaccination is not compulsory although LSD is a notifiable disease. There are currently three companies in South Africa that produce LSDV vaccines, two of the vaccines contain cell‐adapted strains of the original LSDV Neethling strain. It is not clear if the vaccine strains have been molecularly characterized using whole genome sequencing but both of these vaccines have been confirmed to be LSDV using a CaPV species‐specific PCR method (E. Tuppurainen, unpublished data). The third company is using an attenuated South African LSDV field isolate.

Over a 2‐year period, one of the largest cattle feedlot companies in South Africa vaccinated more than 200 000 cattle using the attenuated LSDV field strain vaccine. Then due to financial reasons, the vaccine was changed and over the past 2 years about 150 000 cattle have been vaccinated with the LSDV Neethling strain vaccine. Cattle were vaccinated on arrival to the farm and 14 days later. They were between six and 9 months of age, with an average weight of 230 kg. No side effects were observed after the use of either of the vaccines, except about 20% of animals showed a swelling at the site of inoculation, which disappeared after a few days. No vaccine breakdowns were detected in these herds but as the exposure of cattle to LSDV was not known, the effectiveness of vaccination in a feedlot was difficult to determine (Personal communication Dr D. Verwoerd, veterinarian).

Notwithstanding the availability of effective vaccines, a large number of outbreaks are still occurring in South Africa. Outbreaks are normally small, involving five to ten herds, with only a few animals within the herd showing clinical signs. Considering the high numbers of outbreaks, and the fact that the disease has a low mortality, one can only speculate that many farmers in South Africa do not regularly vaccinate their cattle against LSDV.

In a previous review, the authors indicated that when the incidence of LSD was low, vaccine use dropped to low levels and therefore over a number of years there has not been sustained use of LSD vaccines. Annual sales have seldom risen above two million doses over a period of 15 years, with a coverage of roughly 20% of the cattle population in South Africa in 2000. These authors also showed that the Neethling vaccine strain cross‐neutralized LSDV field strains (Hunter and Wallace, 2001). The number of vaccine doses sold per year by the different companies is not available.

The attenuated South African vaccine strain has been shown to protect against clinical disease, but experiences during the outbreaks in 1990/91 challenged the assertion that immunity to LSD is life‐long, and annual vaccination is now recommended. Investigations following reports of ‘vaccine breakdown’ are not consistent with a lack of vaccine efficacy. ‘Vaccine breakdown’ has been linked to vaccination of animals that were already incubating the disease, confusion of the disease with ‘pseudo lumpy skin’ disease (Allerton virus, BHV‐2) or LSDV infection in unvaccinated calves, after the disappearance of maternal antibodies (Hunter and Wallace, 2001).

According to one South African manufacturer, recently observed adverse vaccine reactions in the field were associated with the increased susceptibility of European high‐producing cattle breeds or lack of appropriate needle hygiene. A skin reaction caused by the vaccine virus at the injection site was detected in 2–10% of animals as well as slight reduction in milk production for 4–5 days.

During the 2006 outbreak of LSD in Egypt, it was reported that the live attenuated KSGP O‐240 strain did not provide cattle with complete protection against LSD (Salib and Osman, 2011). Incomplete protection was also observed when the RM65 SPP vaccine was used to vaccinate cattle against LSDV in Israel at the same dose as for sheep from 2006 to 2007 (Brenner et al., 2009).

In a randomized controlled field study, safety and efficacy of LSDV and RM65 (as a ten times higher dose as for sheep) vaccines were compared in dairy and beef cattle. Two to 5 months prior to the onset of the study, these animals were vaccinated with a single dose of RM65 vaccine using the sheep dose. Both LSDV and RM65 (10X) vaccines were safe to use, mild adverse effects were detected after vaccination using the LSDV vaccine. The efficacy of LSDV vaccine was superior when compared with the RM65 SPPV (10X) vaccine (Ben‐Gera et al., 2015).

Recently several reports have been published reporting LSD vaccine failure in Ethiopia. The LSDV Neethling and KSGP O‐180 strain vaccines, both produced locally by the National Veterinary Institute (NVI) are used in cattle against LSDV in Ethiopia. In 2008 and 2009 re‐infection of vaccinated animals was observed during LSDV epidemics. The highest morbidity (15.1%) and mortality (5.37%) of LSD were observed in vaccinated feedlot cattle rather than in extensively managed cattle (Ayelet et al., 2014). Another study in Ethiopia reported morbidity and mortality rates of 22.9% and 2.31% respectively in fully vaccinated herds (Ayelet et al., 2013). Similar vaccine failure has been reported in sheep vaccinated against SGPV using the NVI KSGP O‐180 vaccine (G. Gari, unpublished data), highlighting the need for molecular characterization of the vaccine seed viruses and re‐assessment of the level of attenuation of the local vaccines.

Recently, Gari et al. (2015) compared the efficacy and immunogenicity of NVI LSDV Neethling and KSGP O‐180 strain vaccines and the Gorgan GTP strain vaccine (Caprivac^™^, Jordan Bio‐Industries Center, Amman, Jordan) produced by the Jordan Bio‐Industries Centre (JOVAC). The study included vaccine challenge experiments in a controlled environment and monitoring of immune responses in vaccinated animals in the field. The Ethiopian Neethling and KSGP O‐180 vaccines failed to provide protection in cattle against LSDV, whereas the Gorgan GTPV vaccine protected all the vaccinated calves from clinical signs of LSD. Moreover, the Gorgan GTPV vaccinated cattle showed higher levels of cellular immune responses at the vaccination site, consistent with greater immunogenicity (Gari et al., 2015).

### Ideal vaccine product profile

An ideal vaccine would provide rapid onset of lifelong humoral and cell mediated immunity within 14 days of a single administration. The vaccine should be safe and not cause clinical disease or spread to non‐vaccinated animals. In addition, the vaccine should be inexpensive and thermostable.

A single vaccine against SPP, GTP and LSD would be ideal (Kitching, 2003) and is technically feasible. Recombinant vaccines, which use SPPV, GTPV or LSDV as a vaccine vector, may however face regulatory issues in countries that do not have all three diseases. For example, a SPPV or GTPV derived vaccine would not be used in South Africa, and a LSDV derived vaccine would not be used in Asian countries. Within the European Union (EU), eradication of CaPV is in general based on total stamping‐out of all infected and in‐contact animals, animal movement restrictions and other supporting eradication measures. However, use of emergency vaccinations may be allowed if it would not affect the interests of the other EU member states (92/119/EEC of 17 December 1992).

For non‐endemic countries, a DIVA vaccine needs to be developed. This vaccine would also be a useful tool for endemic countries to eventually acquire disease‐free status following the implementation of an effective eradication campaign.

Killed CaPV vaccines are safe to use in non‐endemic countries in emergency scenarios although more than a single administration is required. Currently, the efficacy of killed vaccines against LSD, SPP and GTP viruses are under re‐evaluation. To date, no commercially available viral vectored vaccines using CaPV antigens have been developed.

### Previous research on recombinant vaccines for SPP, GTP and LSD

Due to its large genome LSDV has been used as a vaccine backbone for many viruses. When the fusion (F‐protein) (Romero et al., 1994), as well as the hemagglutinin (H‐protein) genes (Romero et al., 1994) of the rinderpest virus were generated in two separate constructs, utilizing the thymidine kinase (TK) region of the KS‐1 virus (LSDV), both recombinant viruses were able to protect cattle from rinderpest as well as LSD. The KS‐1 vaccine expressing the H‐protein was evaluated in cattle to determine the duration of immunity and the vaccine was able to protect 100% of cattle against LSDV and 50% of the cattle against rinderpest following an experimental challenge 2 years after vaccination (Ngichabe et al., 2002). The KS‐1 vaccine expressing the rinderpest F‐protein was also able to protect goats against PPR virus (Romero et al., 1995).

When the F (Diallo et al., 2002) or H genes (Berhe et al., 2003) of PPR virus were inserted in KS‐1 vaccines in the TK region, both vaccines were able to protect against lethal PPR challenge. Another study examined the role of pre‐existing CaPV immunity on immunity generated by the KS‐1 containing the PPR F gene. This study indicated that pre‐existing CaPV immunity led to partial protection against PPR virus (Caufour et al., 2014). The F gene or H gene from PPR virus inserted into the TK region of GTPV vaccine AV41 was able to induce PPR virus specific antibodies in sheep and goats as well as protection against GTPV in goats (Chen et al., 2010). This study further demonstrated that two immunizations were able to overcome pre‐existing immunity. Antigens from bluetongue virus (BTV), including VP7 (Wade‐Evans et al., 1996), VP2, NS1 and NS3 (Perrin et al., 2007), have also been generated in KS‐1, and have demonstrated partial protection against BTV challenge in sheep and goats. Rift Valley fever virus (RVFV) glycoproteins Gn and Gc expressed in KS‐1 have been able to elicit RVFV neutralizing antibodies and protection against RVFV in sheep (Wallace et al., 2006; Soi et al., 2010). Collectively, these studies demonstrate the utility of CaPV as a vaccine vector.

There have been limited studies regarding the attenuation of CaPV through gene knockout. The virus Kelch‐like gene SPPV‐019 was demonstrated to attenuate a virulent SPPV, and this attenuated virus was able to provide protection against challenge with virulent SPPV infection in sheep (Balinsky et al., 2007). It is likely that there are multiple genes which could be disrupted in CaPV that can attenuate the virus and possibly improve the immunogenicity of the vector.

### On‐going research using CaPVs as vaccine vectors

There are many different genes that could potentially be used to attenuate CaPV. This is illustrated by the numerous attenuated CaPV vaccines that have been generated through multiple passages in cell culture, each with a variety of different mutations. Using homologous recombination, a LSDV knock‐out of a virulence gene has been generated and this virus is partially attenuated in cattle and fully attenuated in sheep and goats (S. Babiuk, unpublished results). Additional gene knockouts in this attenuated LSDV are being generated to fully attenuate this virus in cattle (S. Babiuk, unpublished results). Using this attenuated LSDV the protective antigens from PPR virus (F antigen) and RVFV (glycoproteins) are being evaluated in sheep and goats (Boshra et al., 2013). In addition, a LSDV attenuated vaccine with RVFV antigens is being developed for use in South Africa. Thus LSDV vectors can be used as a vaccine platform with the flexibility to include the vaccine antigens which are required for different regions. A similar approach can be used to attenuate SPPV and GTPV viruses to allow countries with these viruses to develop vaccines which satisfy their regulatory agency’ policies. Furthermore, once a validated ELISA becomes available, it is a possibility that a DIVA vaccine could be developed by knocking out the gene that encodes the ELISA antigen in the vaccine. However, to do this would require that the ELISA antigen would be encoded by a non‐essential highly immunogenic gene for CaPV to guarantee an immune response in infected animals.

### Comparison of the efficacy of the currently used live vaccines and novel inactivated vaccines

Disease‐free countries would hesitate to use live CaPV vaccines on safety grounds and due to the ramifications for international trade restrictions. Two novel inactivated vaccines derived from the LSDV Neethling strain and Romanian SPPV strain have been recently developed by MCI Sante Animale, Morocco. The first challenge experiment, testing the inactivated SPPV and GTPV vaccine for sheep and goats against a virulent field isolate has been carried out by the manufacturer and the field experiments will follow (Z. Boumart, unpublished data). Independent challenge experiments using the killed LSDV Neethling and Romanian SPPV strain vaccines against LSDV in cattle are on‐going by the scientists at CODA CERVA, Belgium (K. De Clercq, unpublished data). The project aims to evaluate safety and efficacy provided by the newly developed inactivated LSDV and Romanian SPPV vaccines for cattle against LSDV, and to compare the performance of the killed vaccines with the efficacy of the following live attenuated, commercially available vaccines: LSDV Neethling, RM65 SPPV (×10), Gorgan GTPV and Bakirköy SPPV (×4) strains. The availability of safe and effective, non‐replicating vaccines would enhance the preparedness of non‐endemic countries for an incursion of CaPVs and provide safer means to control CaPV than live vaccines. However, the use of inactivated vaccines should be considered a short term solution in an emergency (Tuppurainen and Oura, 2014) as the protection provided by inactivated vaccines may not be long‐lasting and booster vaccinations would need to be administered every 6–12 months (Kitching, 1986a).

### DIVA vaccines and companion tests

Currently available CaPV vaccines are derived from field isolates that have been attenuated by serial passage in cell culture, or in cell culture followed by chohrio‐allantoic membranes of embryonated chicken eggs. Only three attenuated vaccine strains (one each derived from a SPPV, GTPV or LSDV field isolate) have been characterized by full‐length genome sequencing. When compared with virulent wild type viruses, a range of genetic changes, including mutation or disruption of genes with predicted functions involving virulence and host range were evident (Tulman et al., 2002; Kara et al., 2003). However, despite providing robust protection, the antibody response elicited in sheep, goats and cattle cannot be distinguished from that in animals which are infected naturally with wild type CaPVs. In an endemic situation, or during and following an outbreak in previously disease‐free regions or countries, this makes it exceedingly difficult, if not impossible, to undertake effective serosurveillance in support of disease control and eradication activities, and thereby establish or re‐establish freedom from disease. One potential solution, conceptually conceived almost three decades ago (Van Oirschot et al., 1986) and which has been progressively applied to a growing number of viral diseases that pose a serious threat to animal health (Pasick, 2004; Vannie et al., 2007; Uttenthal et al., 2010) would be to develop a negative marker vaccine and companion serological assay. In the simplest form of a DIVA vaccine, a live‐attenuated CaPV isolate would be engineered to lack a non‐essential, immunodominant viral antigen that would enable the detection of only infection‐specific antibodies in a susceptible host species, using a companion diagnostic test such as an ELISA. This would enable the differentiation of infected from vaccinated animals, as well as the identification of vaccinated animals which may subsequently have become infected, meaning that vaccination could be used in disease control programs without masking the serological detection of infected animals.

Although gene deletion and insertion in CaPVs is technically relatively straightforward (Wallace and Viljoen, 2005; Wallace et al., 2007; Boshra et al., 2013), identification of a single, suitable, immunodominant marker antigen may prove to be a more significant challenge. Several antibody detection ELISAs, based on recombinant CaPV proteins, have been previously developed. These have used either the mature virion envelope protein P32 (Carn et al., 1994; Heine et al., 1999; Bhanot et al., 2009) or viral core proteins 095 and 103 (Bowden et al., 2009) as coating antigen. However, none of these assays has been shown to be sufficiently sensitive for undertaking large scale, high‐throughput serodiagnosis in sheep, goats and cattle.

### Field evaluation of vaccine efficacy

Vaccines may protect against disease, infection, infectiousness or carriage. As a consequence, protection may be direct to the individual being vaccinated or indirect through a reduced transmission risk which influences the overall effectiveness at the population level (Halloran et al., 1999). Vaccine efficacy describes the total protective effect to vaccinated individuals. In human studies, it is typically defined as the percentage reduction in risk of a defined outcome comparing vaccinated and unvaccinated groups, assuming equal exposure (Halloran et al., 1991). Among veterinary vaccines a definition is less consistently defined and it has been recommended that terminology should be standardized (Knight‐Jones et al., 2014).

Efficacy for veterinary vaccines is typically determined by challenge studies which, due to cost and ethical implications, often use a relatively small number of animals. The method of challenge may not accurately reflect how exposure occurs in the field and the small number of animals leads to statistical uncertainty of the results. Seroconversion studies are commonly performed when evaluating veterinary vaccines using larger groups of animals although field derived correlates of protection are often scarce. Randomized controlled trials are typically used in these efficacy studies for licensure (Knight‐Jones et al., 2014).

Although a vaccine may be shown to be efficacious, this does not necessarily mean that it will perform well when used in a vaccination programme. Reasons for poor performance include inadequate coverage, poor maintenance of the cold chain, incorrect schedules, impact of maternal immunity and antigenic mismatch between the circulating and vaccine strains. ‘Vaccine effectiveness’ is calculated in an identical way to efficacy although under programmed conditions. Observational studies are often used to estimate effectiveness, so it is important that adjustments for confounders are made in the analysis. Generally such studies should be performed at the individual animal level due to a difficulty in accounting for different exposures and different population structures when comparing groups (Knight‐Jones et al., 2014). Cluster (or ‘pseudo‐cluster’) randomized trials comparing areas with or without a control programme are a powerful study design for vaccine evaluation when indirect protection is likely to be important (Vaucher, 2009). Post‐licensure effectiveness studies are extensively performed for human vaccines but are lacking for most vaccines used in animal health.

For CaPVs, there have been numerous reports of vaccine performance in the field (Brenner et al., 2009; Ayelet et al., 2013; Abutarbush et al., 2016). A recently published randomized field study provided evidence that LSDV vaccine was superior to RM65 (10X) in cattle against LSDV in Israel (Ben‐Gera et al., 2015). Calculation of efficacy for either vaccine was not possible in this study due to the absence of unvaccinated controls although field evidence for the superiority of a vaccine is an important information. There is a further need for continued rigorous vaccine effectiveness studies to inform and optimize control strategies. Cluster randomized trials may be difficult to perform due to the difficulty in maintaining independence of the clusters. Moreover, the current absence of a consistent serological correlate of protection makes post‐vaccination monitoring based on serology an unlikely indicator of effectiveness. Therefore, observational studies using a clinical‐based outcome should be performed and protocols put in place to investigate apparent vaccine failures as part of a holistic CaPV control plan.

### Genomic database for poxviruses

Although many 20^th^ century anti‐viral vaccines were developed without the benefit of genomic sequences, current vaccine design strategies depend upon detailed knowledge of the viral genomes.

Such information is essential because it allows researchers to (i) determine the genetic makeup of the current 1st generation vaccines, (ii) analyse the conservation of genes among the circulating LSDV, SPPV and GTPV pathogens and (iii) establish limits of variation for the potential vaccine antigens, i.e. what range of epitopes a vaccine needs to cover. In light of this, a significant component of any future effort to make novel CaPV vaccines will involve the sequencing of numerous CaPV genomes.

CaPVs are relatively large viruses; their dsDNA genomes are approximately 150 kb and they encode about 150 proteins (Tulman et al., 2001, 2002). To date, of the 180+ poxvirus genomes that have been sequenced, only eight are CaPV (Tulman et al., 2001, 2002; Kara et al., 2003); thus, even if only a relatively minor survey of the SPP, GTP and LSD viruses in current circulation is deemed necessary, the number of complete CaPV genomes that must be managed will grow many fold. Thus, the analysis of these viral genomes will require a database system to manage the genomic data (genome/gene/protein sequences) and specialized bioinformatics tools to perform the required comparative analyses.

The Viral Bioinformatics Resource Center (www.virology.ca) managed by Dr Chris Upton at the University of Victoria was created specifically to work with large DNA viruses and focuses on the family *Poxvirus*. This resource is ‘virologist‐centric’, in that it is designed to be used by virologists with little experience in the computer science side of bioinformatics. Thus, it provides relatively easy access to a database of genomes, genes, proteins, promoters and relational information through an easy‐to‐use graphical user interface (GUI). All comparative analyses are performed with similar GUI‐based tools, and results are presented in graphical formats for easier interpretation.

Such a Bioinformatics Resource would provide several important roles for the entire CaPV research community. First, it would lead a community‐wide effort to establish a CaPV genome annotation standard, ensuring genomes are annotated correctly and that a standard is applied to accurately represent fragmented/truncated/overlapping genes. Second, the resource would annotate all newly sequenced genomes (also checking for potential sequencing errors), and third, the resource would train researchers how to take advantage of the specialized tools it provides for performing comparative analyses of CaPV genomes.

### Future research priorities and opportunities

For smallholder farmers and poor rural communities, healthy livestock can provide a cost‐effective and sustainable means to improve lives (Banerjee et al., 2015). Small ruminants are particularly important for development focused on gender inequality, as women of the developing world can play a much greater role in their ownership and production (Gates, 2014). CaPV infections cause significant losses for smallholder farmers and remain a major impediment to the improvement of livestock systems and the development of intensive cattle, sheep and goat production in the Middle East, Africa and Asia. LSD is on the move in the Middle and Near East, causing additional suffering in a region which is already burdened by political unrest, and a growing threat to disease‐free regions, particularly the EU and Asia. LSD, SPP and GTP are currently priority neglected, tropical diseases. Notably, when compared with a number of other neglected diseases important to poverty reduction or to global trade, tools for control do exist and there are feasible options for improved CaPV control (Perry and Grace, 2009).

The growing threat of CaPV incursions in historically disease‐free regions together with an increasing awareness of the role that livestock can play in achieving development goals in CaPV endemic region, underline the need to address the following knowledge gaps;

1. Direct and indirect transmission of CaPV and the role of various arthropod vectors.
2. Efficacy of the currently used vaccines (addressed using challenge experiments and epidemiological field studies).
3. Immunological correlates of vaccine protection.
4. Neutralizing or immunogenic epitopes for a protective response.
5. Developing novel safe, efficient and affordable DIVA vaccines, including inactivated as well as next‐generation and potentially recombinant vaccines and cell lines for vaccine production.
6. and developing a pen‐side assay for the rapid confirmation of the field diagnosis.

Better understanding of the epidemiology, transmission, immunity, as well as development of effective prophylactic tools for LSD, SPP and GTP will ultimately lead to improved disease control, benefiting small‐scale farmers and supporting the livelihood and wellbeing of women, children and poor rural communities in endemic regions.
